# Driving brand equity in health services organizations: the need for an expanded view of branding

**DOI:** 10.1186/s12913-018-3679-4

**Published:** 2018-12-14

**Authors:** James K. Elrod, John L. Fortenberry

**Affiliations:** 1 Willis-Knighton Health System, 2600 Greenwood Road, Shreveport, LA 71103 USA; 2 LSU Shreveport, 1 University Place, Shreveport, LA 71115 USA

**Keywords:** Branding, Brand equity, Identity management, Marketing, Hospitals, Healthcare

## Abstract

**Background:**

Branding—the assignment of names, logos, slogans, and related elements of identity to institutions and their product offerings for the purpose of conveying desired images to target audiences—is of paramount importance in the health services industry. Associated initiatives traditionally have centered on developing verbal and visual brand expressions, but opportunities abound to drive brand equity by supplementing traditional pursuits with new, different, and unexpected expressions that afford highly memorable experiences.

**Discussion:**

Willis-Knighton Health System has possessed an expanded view of branding for decades. While the system has directed thorough attention toward traditional brand expressions, additional identity opportunities outside the bounds of traditional branding thought have been pursued vigorously. There perhaps is no better illustration of Willis-Knighton Health System’s expanded approach to branding than that of Willis the Bear, the institution’s iconic teddy bear mascot developed to promote labor and delivery services. This article presents the origins and development of this brand expression, particularly emphasizing the need to address nontraditional elements of identity for purposes of driving brand equity.

**Conclusions:**

Given the importance of brand management and extraction of associated value, health services organizations must diligently direct attention toward branding initiatives. Traditional approaches, when executed well, deliver excellent results, but enhanced value can be derived by addressing nontraditional brand elements which afford unique opportunities to differentiate given establishments from their competitors, facilitating institutional viability and vitality.

## Background

Healthcare providers face an unending barrage of activities and obligations required for the successful delivery of medical services [1, 2]. Many of these pursuits pertain to marketing [3–6] and, of these, one of paramount importance is that of branding, the assignment of names, logos, slogans, and related elements of identity to institutions and their product offerings for the purpose of conveying desired images to target audiences [7, 8]. Aside from presenting imagery designed to resonate with customers, thus enticing them to forward their patronage, branding also permits audiences to distinguish institutions and their products from competitive offerings, making the practice essential for facilitating product differentiation [7, 9–12]. Symbols representing given healthcare establishments, color schemes on associated logos, jingles featured in television commercials, and so on all emerge through branding initiatives. Since branding efforts indeed assign identity to institutions and their product offerings, branding sometimes is referred to as identity management [13–15].

Branding constitutes the very beginning of the marketing communications process [8, 15]. Before advertisements and other forms of promotion are prepared, the imagery associated with the institution and its products must, of course, be developed. As such, great care must be taken to ensure that branding is addressed accurately and comprehensively before advancing communications initiatives [15, 16]. Since branding efforts represent the cornerstone of all marketing communications, neglectful formulation will negatively impact forthcoming advertisements and other forms of promotion, making proper assembly of brands an absolute necessity [7–10]. Once thoughtfully prepared, however, branding efforts do not cease. Instead, they continue indefinitely as a means of ensuring that formulated identities remain meaningful and relevant, something essential due to the ever-changing nature of the environment and its associated circumstances and challenges [15–18]. Brands indeed must evolve as environments evolve. A brand expression relevant today can become irrelevant with the passage of time as tastes, preferences, policies, and other variables change, necessitating vigilance in monitoring brand performance and a willingness to introduce updates and enhancements as needed to ensure excellence.

Branding activities traditionally have centered on preparing verbal (e.g., names, slogans) and visual (e.g., logos, illustrations) brand expressions [7, 16, 19], but today, increasing attention is being directed toward developing branding imprints across the full array of senses—sight (e.g., logos, illustrations, unique color schemes), sound (e.g., signature audio tones before commercials, branded music-on-hold services), taste (e.g., logo-bearing cookies, premium meals for patients), touch (e.g., logo-embossed business cards, signature linens, branded stuffed animals), and smell (e.g., aromatherapy in patient rooms, signature scents within institutions)—as occasions permit [7, 19–22]. Such an expanded view of branding offers opportunities to connect with patients in ways never before imagined. Supplementing traditional brand expressions with new, different, and unexpected expressions affords highly memorable experiences which drive brand equity (i.e., the value of the brand) [8, 15, 20].

**Fig. 1:**
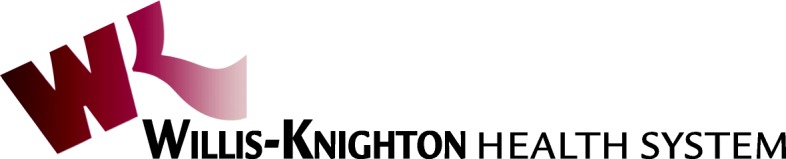
Willis-Knighton Health System’s logo

**Fig. 2:**
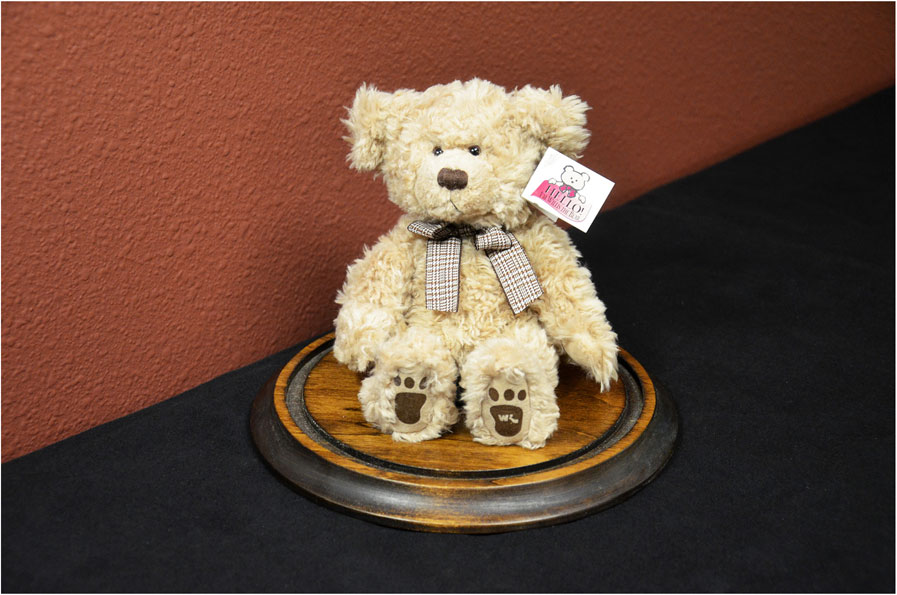
Willis-Knighton Health System’s Willis the Bear

Willis-Knighton Health System has possessed an expanded view of branding for decades, something achieved as a result of the institution’s willingness to experiment and innovate at every possible opportunity [23]. While the system has directed thorough attention toward traditional brand expressions, notably achieving one of the most widely recognized logos in the marketplace (as presented in Fig. 1) [24], additional identity opportunities outside the bounds of traditional branding thought have been pursued vigorously. There perhaps is no better illustration of Willis-Knighton Health System’s expanded approach to branding than that of Willis the Bear, the institution’s iconic teddy bear mascot developed to promote labor and delivery services. Presented in Fig. 2, this particular brand manifestation falls well outside of traditional branding pursuits, but carries significant identity value, magnifying the institution’s collective branding efforts and aiding in the establishment of a fruitful brand experience for patients.

## Discussion

In the mid-1990s, after many years of concerted effort, Shreveport, Louisiana-based Willis-Knighton Health System assumed market leadership in the Ark-La-Tex, the region of America where the states of Arkansas, Louisiana, and Texas converge. With construction of its newest hospital, WK Pierremont Health Center, beginning soon, the stage was set for the institution to serve an even greater number of patients, further advancing Willis-Knighton Health System’s market position. Maternity services, in particular, witnessed dramatic growth during this era, with an increasing number of women turning to Willis-Knighton Health System for their labor and delivery needs. This uptrend had begun years earlier, courtesy of a series of strategic investments, including the employment of highly-skilled caregivers, provision of the region’s most advanced medical technologies, delivery of care in attractive and inviting medical servicescapes featuring appealing labor, delivery, and recovery (LDR) rooms, and initiation of unique target marketing strategies. Associated services, carrying Willis-Knighton Health System’s carefully crafted brand identity, were marketed effectively via advertisements and other forms of promotion, attracting mothers-to-be in high numbers, with each being drawn to the institution’s well-rounded service experience designed exclusively for expectant moms.

With traditional branding initiatives addressed comprehensively, Willis-Knighton Health System investigated possible opportunities to expand branding efforts to bolster identity and add a degree of uniqueness to the labor and delivery experience. During this particular era, new mothers remained hospitalized for several days following childbirth, leading executives to consider offering something special for the new parents while they awaited returning home. Ultimately, a steak dinner, served either in the hospital’s private dining room or bedside, was chosen, providing a premium meal for the parents to enjoy in celebration of the birth of their baby. This novel brand expression delivered extensive value for many years, but in the late 1990s, health insurance reimbursement changes were introduced, ushering in revised protocols which necessitated that new mothers be discharged shortly after delivering their babies. Quite obviously, this alteration demanded modification of many service delivery elements, including the steak dinner brand expression. With discharge occurring so quickly after delivery, the dinner, by necessity, had to be boxed up and sent home with the new parents, relegating the formerly meaningful experience to nothing more than a carry-out meal offering little brand value. With the steak dinner brand expression diminished, a worthy successor was sought.

Over the Christmas 1999 holiday season, a fitting replacement emerged, courtesy of an idea elicited by a store display at a local shopping mall. A particular retailer happened to be offering a custom teddy bear, prompting the notion that a Willis-Knighton Health System-branded teddy bear might serve as a suitable brand expression for the institution’s labor and delivery services. A teddy bear was purchased and the concept was vetted comprehensively, drawing on perspectives from within and outside of the organization, in an effort to ascertain branding potential and value. Ultimately, the idea was deemed to be meritorious, with the branded teddy bear being viewed as unique (i.e., not offered by any healthcare provider in the market), memorable (i.e., expected to leave a lasting impression), appropriate (i.e., viewed as a perfect gift to celebrate a child’s birth), enduring (i.e., expected to be kept and appreciated), and marketable (i.e., viewed to possess significant promotions potential). Such qualities, well known to be attributes of excellent brands [25, 26], gave Willis-Knighton Health System confidence in the viability of the concept.

**Fig. 3:**
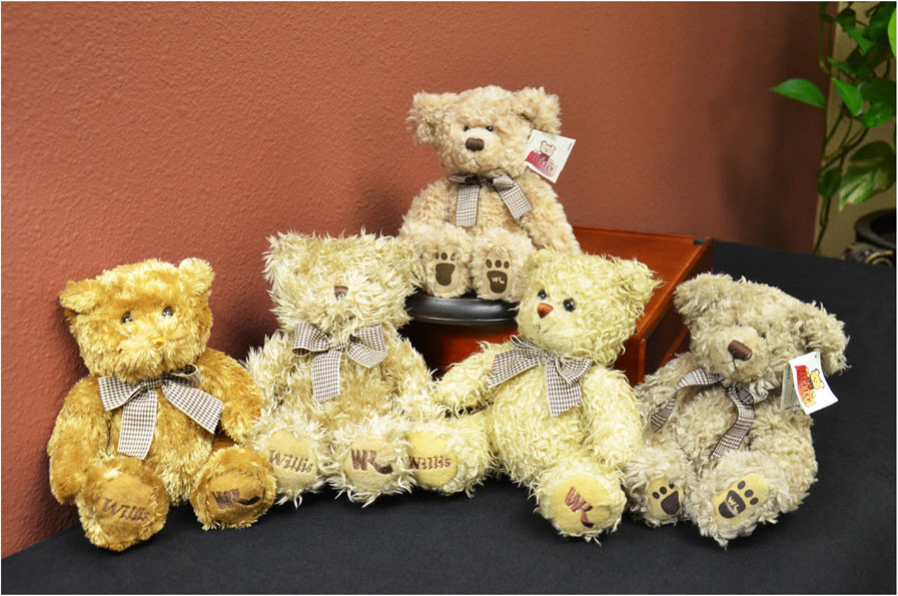
Willis-Knighton Health System’s Willis the Bear (top) and several prototypes

Financial feasibility was next assessed and, for this, Willis-Knighton Health System reached out to the company that produced the sample bear purchased at the mall, inquiring regarding the ordering process, options, minimum order quantity, costs, production dynamics, and so forth. On learning of these details and determining that they were acceptable to the institution, the project was approved and initiated. Working with representatives from the teddy bear manufacturer, staff members evaluated a range of options (e.g., size, type and color of fur, wardrobe, logo presentation opportunities, etc.) and carefully selected associated attributes. As presented in Fig. 3, a number of prototypes were developed (i.e., the four bears seated on the table’s surface), permitting executives to view design choices firsthand, with this process ultimately yielding the finished product (i.e., the bear seated on the pedestal). Known as Willis the Bear, Willis-Knighton Health System’s teddy bear stood 8 in. tall and featured tan fur, a houndstooth bow placed around his neck, and an introduction tag attached to his ear. Significantly, especially for purposes of enduring promotional value, Willis-Knighton Health System’s logo was stitched into Willis the Bear’s paw, ensuring that, even with lots of use and abuse, brand identity would remain intact. With specifications determined, Willis-Knighton Health System’s order was placed and in a matter of weeks the institution received a container loaded with the custom teddy bears. Willis the Bear made his official debut on Mother’s Day 2001.

Since its introduction, over 64,000 Willis the Bear stuffed animals have been distributed, one for every baby born at Willis-Knighton Health System, commemorating the precious event of childbirth. To ensure that the expression retains its exclusivity, the bears are made available only to those who deliver their babies at a Willis-Knighton Health System hospital. They are not offered in gift shops or available via any other route. This particular approach to distribution has hastened Willis the Bear’s almost legendary status. In fact, staff members charged with distributing them routinely are bombarded with inquiries from fellow employees, patients for services other than labor and delivery, and even members of the general public, inquiring as to how they can receive one of the stuffed animals. The bears initially were stored in a locked supply room and distributed as needed to each of Willis-Knighton Health System’s hospitals, cross-checking births against bears delivered to maintain inventory control, but later, the decision was made to place the bears in the institution’s Pyxis systems (i.e., automated medication dispensing units) for distribution convenience and security. To this day, expectant mothers regularly ask staff members if they will receive a bear, indicating how well Willis the Bear works as a brand expression.

**Fig. 4:**
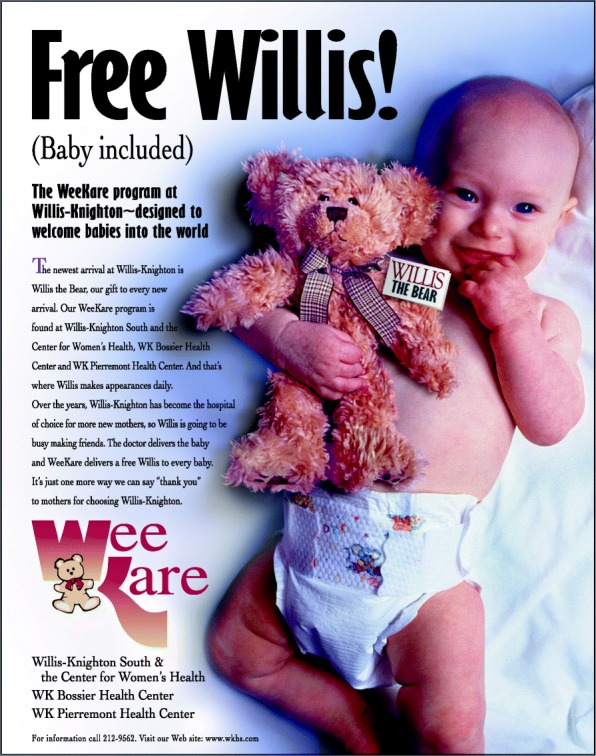
An advertisement featuring Willis-Knighton Health System’s Willis the Bear

**Fig. 5:**
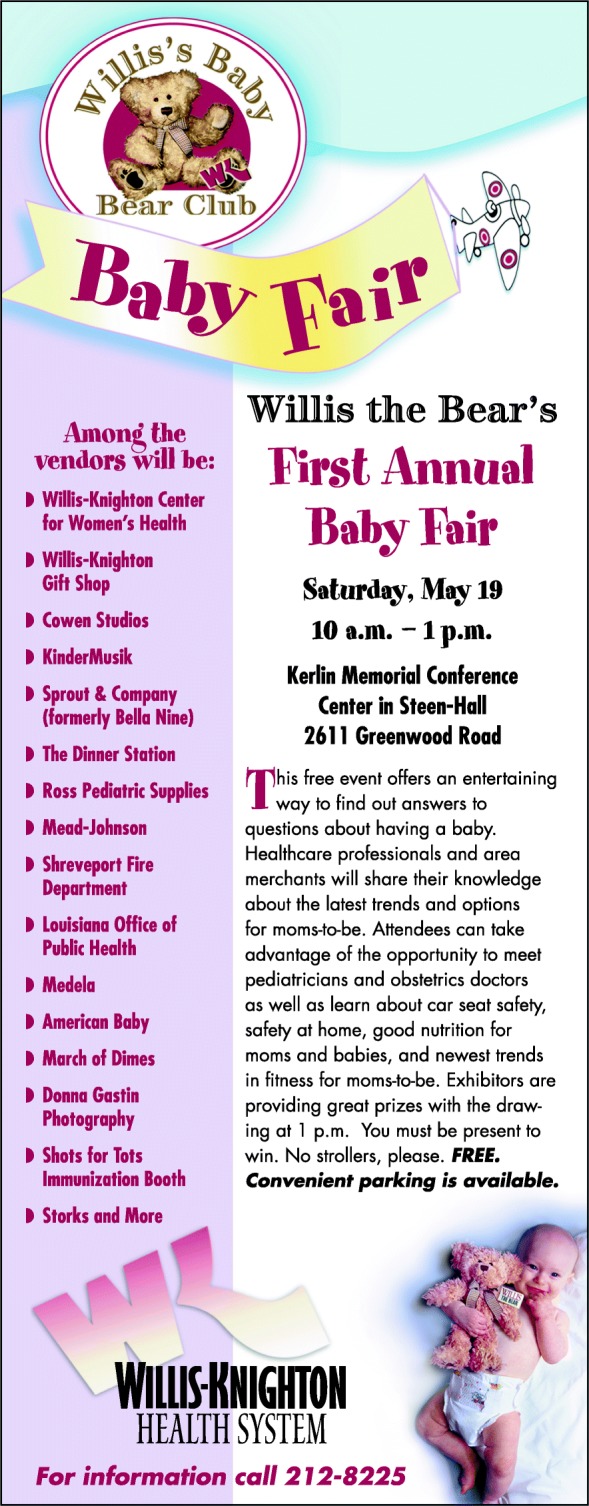
An advertisement promoting Willis the Bear’s Annual Baby Fair

From its inception, Willis the Bear has more than lived up to its expectations as a valued brand expression. Beyond perhaps the most important result—that Willis-Knighton Health System’s labor and delivery customers love the bears—the marketing utility afforded by the stuffed animals has been immense. As presented in Figs. 4 and 5, Willis the Bear has appeared in advertisements promoting Willis-Knighton Health System’s labor and delivery services and he has served as an excellent promotional anchor giving identity to related offerings, including WeeKare, Willis’s Baby Bear Club, and Willis the Bear’s Annual Baby Fair, which provide educational resources for expectant mothers. Evidence suggests that the associated investment has been worthwhile, generating attention and interest far surpassing expectations. Of particularly significant intrigue, there are accounts from parents who delivered their babies years ago who note that their children, many now as old as teenagers, still have their Willis the Bear stuffed animals, some of which were noted to be on display in their households. As Willis the Bear recipients enter adulthood and begin families of their own, executives fully expect decisions regarding their caregiver of choice to be positively influenced by Willis the Bear, with this representing perhaps the highest fulfillment possible for the brand expression.

## Conclusions

Willis-Knighton Health System’s Willis the Bear mascot illustrates the power and utility of supplementing traditional branding pursuits with nontraditional, expanded perspectives. A simple little stuffed animal has been shown to deliver robust value, enhancing identity management efforts system wide, ultimately fostering brand equity. From a Willis-Knighton Health System logo being stitched into his paw (which ensures that brand identity remains in place) to presentation of the character in the form of a stuffed animal (which supports its open and ongoing display in homes) to restrictive distribution (which ensures exclusivity), Willis the Bear was carefully devised to derive maximum benefits from the associated brand expression. While such expanded branding manifestations do not replace traditional verbal and visual brand expressions, if well crafted, they create marketing communications synergies which magnify the totality of identity management efforts.

Arriving at brand expressions falling outside of traditional parameters requires creativity and a willingness to explore and experiment with unique possibilities, coupled, of course, with an intimate understanding of patients and their associated wants and needs. While intensive efforts are required to identify worthy nontraditional brand expressions, on discovery and realization, true brand assets emerge, fortifying the brand portfolios of given healthcare establishments. As with all branding initiatives, vigilance is required, as environmental changes can erode the value of given expressions, just as Willis-Knighton Health System experienced when reimbursement changes diminished the impact of its steak dinner brand expression. By directing ongoing attention toward branding initiatives in the context of the larger environment, however, any required alterations or replacements can be addressed in a timely fashion to ensure continued branding prowess, leading in some situations, as in the case of Willis the Bear, to even greater brand value. Given that institutional viability and vitality hinge, at least in part, on branding successes, associated identity management activities should not be taken lightly by healthcare establishments. An expanded approach to branding which taps nontraditional elements serves as the perfect complement to traditional pursuits, magnifying the potential of health and medical institutions to successfully attract and engage their patient populations.
